# Data on draft genome sequence of *stenotrophomonas* sp. SAM-B isolated from a mineral cold spring located in Tyva, southern Siberia

**DOI:** 10.1016/j.dib.2020.106278

**Published:** 2020-09-04

**Authors:** Elena S. Kashkak, Vladimir Ya Kataev, Yuri A. Khlopko, Valentina G. Budagaeva, Erzhena V. Danilova, Urana S. Oorzhak, Olga P. Dagurova, Andrey O. Plotnikov

**Affiliations:** a The Department of Chemistry, Tuvan State University, 36 Lenin St., Kyzyl 667000, Russian Federation; b Institute for Cellular and Intracellular Symbiosis, Ural Branch of Russian Academy of Sciences, 11 Pionerskaya St., Orenburg 460000, Russian Federation; c Institute of General and Experimental Biology, Siberian Branch of Russian Academy of Sciences, 6 Sakhyanovoy St., Ulan-Ude 670047, Russian Federation

**Keywords:** *Stenotrophomonas*, Mineral cold spring, Whole-genome sequencing, Illumina

## Abstract

*Stenotrophomonas* sp. SAM-B was isolated from Uzharlyg Mineral Cold Spring, Samagaltay Settlement, Republic of Tyva (Southern Siberia), Russian Federation. A whole genome sequencing of *Stenotrophomonas* sp. SAM-B was performed using an Illumina MiSeq platform. The resulting draft genome contains 4,253,956 bp with 66.48% GC-content and 71 contigs; the longest contig contains 968,648 bp, and the N_50_ has a length of 401,736 bp. The genome includes 3816 protein-coding genes, among which 23 are responsible for protein degradation, 65 are associated with stress response, and 31 are associated with virulence, disease, and defense, including beta-lactamase and resistance to fluoroquinolones. The genome data on the SAM-B strain provides fundamental knowledge that would allow a better understanding of the microorganisms inhabiting cold water environments. Moreover, the results of the genome annotation indicated that diverse metabolic pathways are encoded in the genome of the SAM-B strain and that it has biotechnological potential. The draft genome sequence of *Stenotrophomonas* sp. SAM-B has been deposited in DDBJ/ENA/GenBank under the accession number JABBXB000000000; the accession number of the genome sequence referred to in this paper is JABBXB010000000.

**Specifications Table**

**Table:** 

Subject	Immunology and Microbiology; Genetics, Genomics and Molecular Biology
Specific subject area	Bacterial genomics and phylogenomics
Type of data	Draft genome sequence data, figures, tables
How data were acquired	Whole-genome sequencing on a MiSeq platform (Illumina). The genome was assembled with SPAdes v. 3.14.0, annotated using RAST and PGAAP.
Data format	Raw reads, assembled and analyzed draft genome sequences
Parameters for data collection	Isolation of strain; extraction of genomic DNA from a pure culture; DNA library preparation; whole genome sequencing; de novo assembly; annotation
Description of data collection	Genomic DNA extraction was performed from a pure culture of *Stenotrophomonas* sp. SAM-B using a Quick-gDNA™ MiniPrep Kit; library was prepared using a NEBNext® Ultra™ II FS DNA Library Prep Kit for Illumina®; sequencing was performed using a MiSeq Illumina system. The genome was assembled using SPAdes v. 3.14.0, annotated using RAST and PGAAP.
Data source location	*Stenotrophomonas* sp. strain SAM-B was isolated from Uzharlyg Mineral Cold Spring, Samagaltay Settlement, Republic of Tyva (Southern Siberia), Russian Federation (50.6158N, 94.9610 E)
Data accessibility	A sequence of 16S rRNA gene has been deposited to NCBI GenBank under accession number MT883430.1. Direct link to data: https://www.ncbi.nlm.nih.gov/nuccore/MT883430.1/. Raw reads have been deposited in the NCBI Sequence Read Archive under accession number SRR11585867. Direct link to data: https://www.trace.ncbi.nlm.nih.gov/Traces/sra/?run=SRR11585867. Genome scaffolds have been deposited in DDBJ/ENA/GenBank under accession number: GCA_013387485.1. BioProject number: PRJNA627094, BioSample number: SAMN14651246. Genome assembly and annotated data are available in DDBJ/ENA/GenBank under name: ASM1338748v1. Direct link to data: https://www.ncbi.nlm.nih.gov/Traces/wgs/JABBXB01?display=contigs&page=1. Annotation data are supplemented to the article. Figures and table are accessed in this article.

## Value of the Data

• The genome data of *Stenotrophomonas* sp. SAM-B provides insight that would allow an improved understanding of microorganisms inhabiting cold water environments.
• The genome data of *Stenotrophomonas* sp. SAM-B can be used for metabolic studies wherein various processes, pathways, and biomolecules, including protein biodegradation in cold water environments and proteinases that remain active at low temperatures, may be explored.
• The genome data of *Stenotrophomonas* sp. SAM-B would be useful for comparative genomic studies of the genus *Stenotrophomonas* and can be used to improve the taxonomy of the *Stenotrophomonas* species.

## 1 Data Description

Proteolytic microorganisms, which have protein biodegradation capabilities, are found in different ecosystems, including extreme environments, e.g. soda lakes [1, 2]. It is likely that all microbial communities harbor proteolytic microorganisms [1]. Therefore, proteolytic enzymes produced by microorganisms are of great interest in microbial ecology, which aims to expand our understanding of microorganisms that inhabit various environments, including those in extreme conditions [2]. Moreover, proteinases isolated from microorganisms have been widely used in chemical industries, biotechnology, medicine, and molecular biology [2,3].

**Fig. 1:**
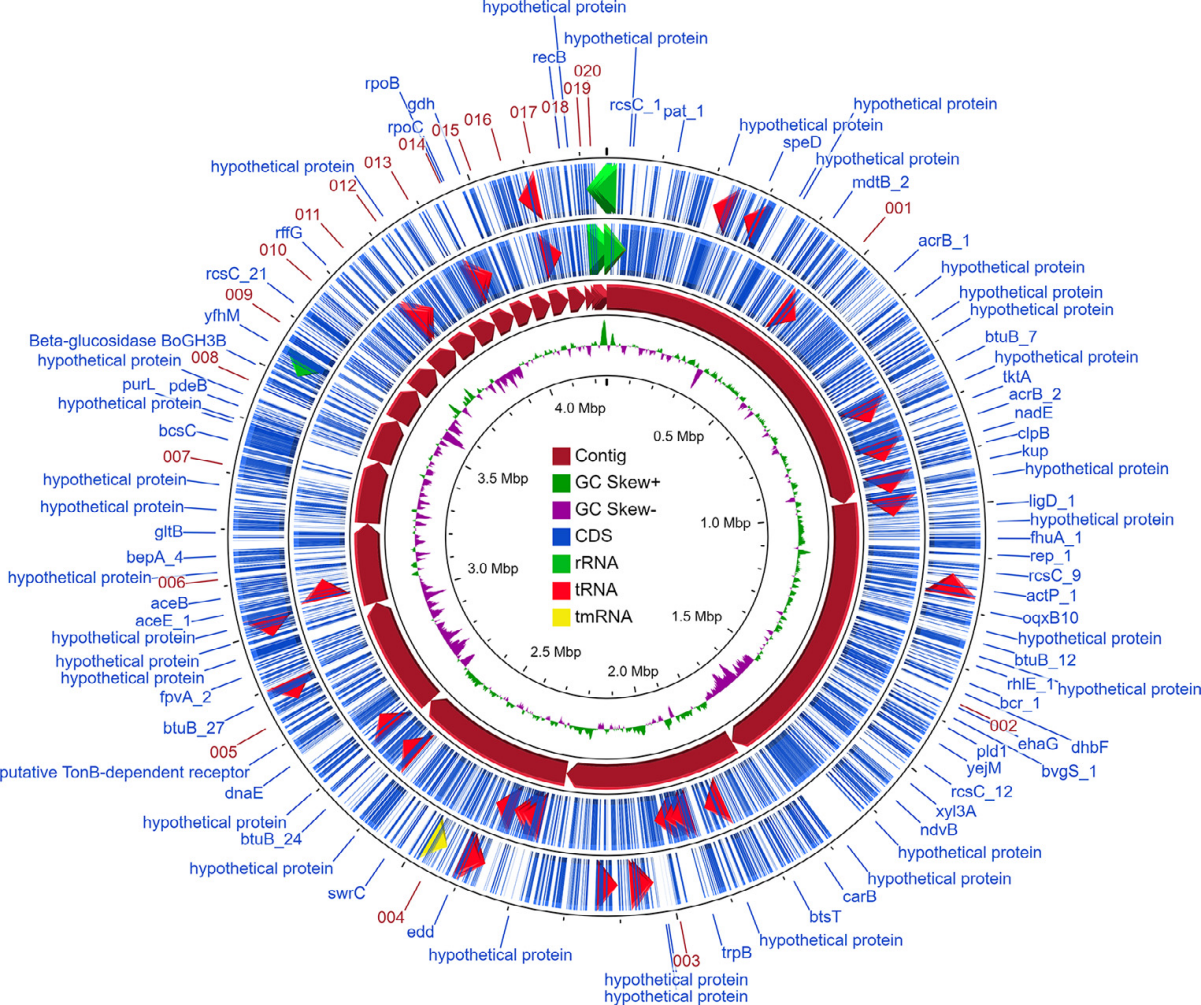
Circular map of the genome of *Stenotrophomonas* sp. SAM-B. Each ring represents the loci of genes that are labeled outside the outermost ring: (from outermost to innermost) forward coding sequences (blue); reverse coding sequences (blue); contigs (dark red); GC skew +/− (green/violet); genome size (black). Triangles within rings: rRNA (green); tRNA (red); tmRNA (yellow).(For interpretation of the references to color in this figure legend, the reader is referred to the web version of this article.)

**Fig. 2:**
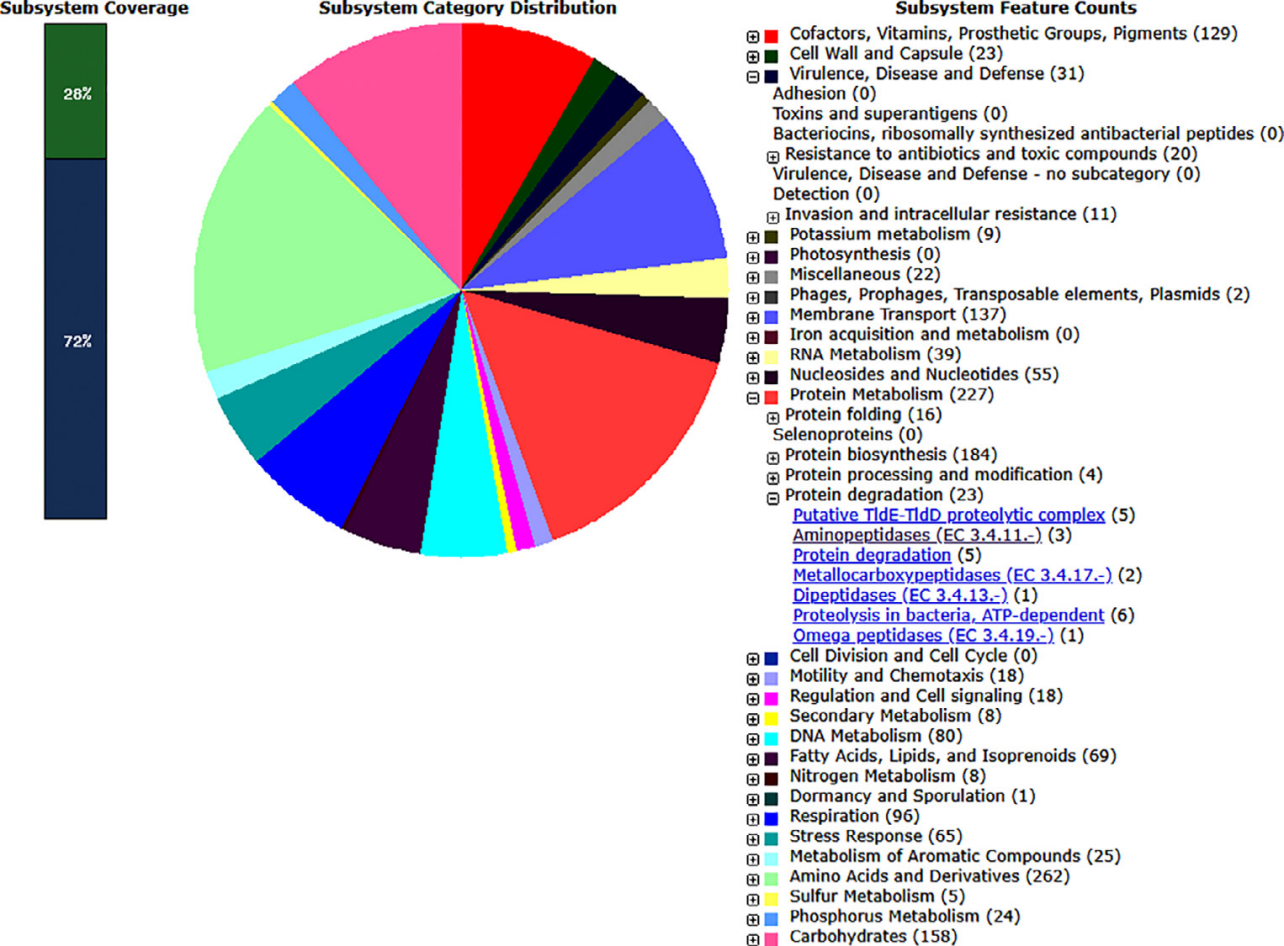
Overview of the subsystem categories assigned to the genome of *Stenotrophomonas* sp. SAM-B. The genome assembly was annotated using the RAST server.

*Stenotrophomonas* sp. strain SAM-B was isolated from Uzharlyg Mineral Cold Spring (Southern Siberia). DNA extraction and whole genome sequencing resulted in a draft genome that was assembled and annotated. Statistics on the assembled genome of *Stenotrophomonas* sp. SAM-B is shown in Table 1. The draft genome contains 4253,956 bp with 66.8% GC-content and 71 contigs; the longest contig contains 968,648 bp, and the N_50_ has a length of 401,736 bp. The genome includes 3816 coding sequences (CDSs) and 113 RNA gene fragments (Supplementary Data). The genome features of *Stenotrophomonas* sp. SAM-B are illustrated in Fig. 1, which was prepared by using CGView Server for genome visualization [4]. The most represented subsystem features that were identified using RAST were amino acids and derivatives (262), protein metabolism (227), carbohydrates (158), membrane transport (137), cofactors, vitamins, prosthetic group, and pigments (129). In the 3816 protein-coding genes of SAM-B strain, 23 were associated with protein degradation, 65 with stress response, and 31 with virulence, disease, and defense, including beta-lactamase and resistance to fluoroquinolones (Fig. 2; Supplementary Data). The data obtained indicated diverse metabolic pathways encoded in the genome of strain SAM-B and significant biotechnological potential. Thus, the SAM-B strain seems promising for use in different biotechnological processes in cold environments, e.g., for bioutilization of waste material or as a source of proteinases that remain active at low temperatures.

**Table 1:** Genome statistics of *Stenotrophomonas* sp. strain SAM-B.

Attribute	Value
Genome size, bp	4253,956
Largest contig, bp	968,648
N50	401,736
L75	5
Attribute	Value
Number of contigs	71
*G* + *C*,%	66,8
Number of Coding Sequences	3816
RNAs	113

**Fig. 3:**
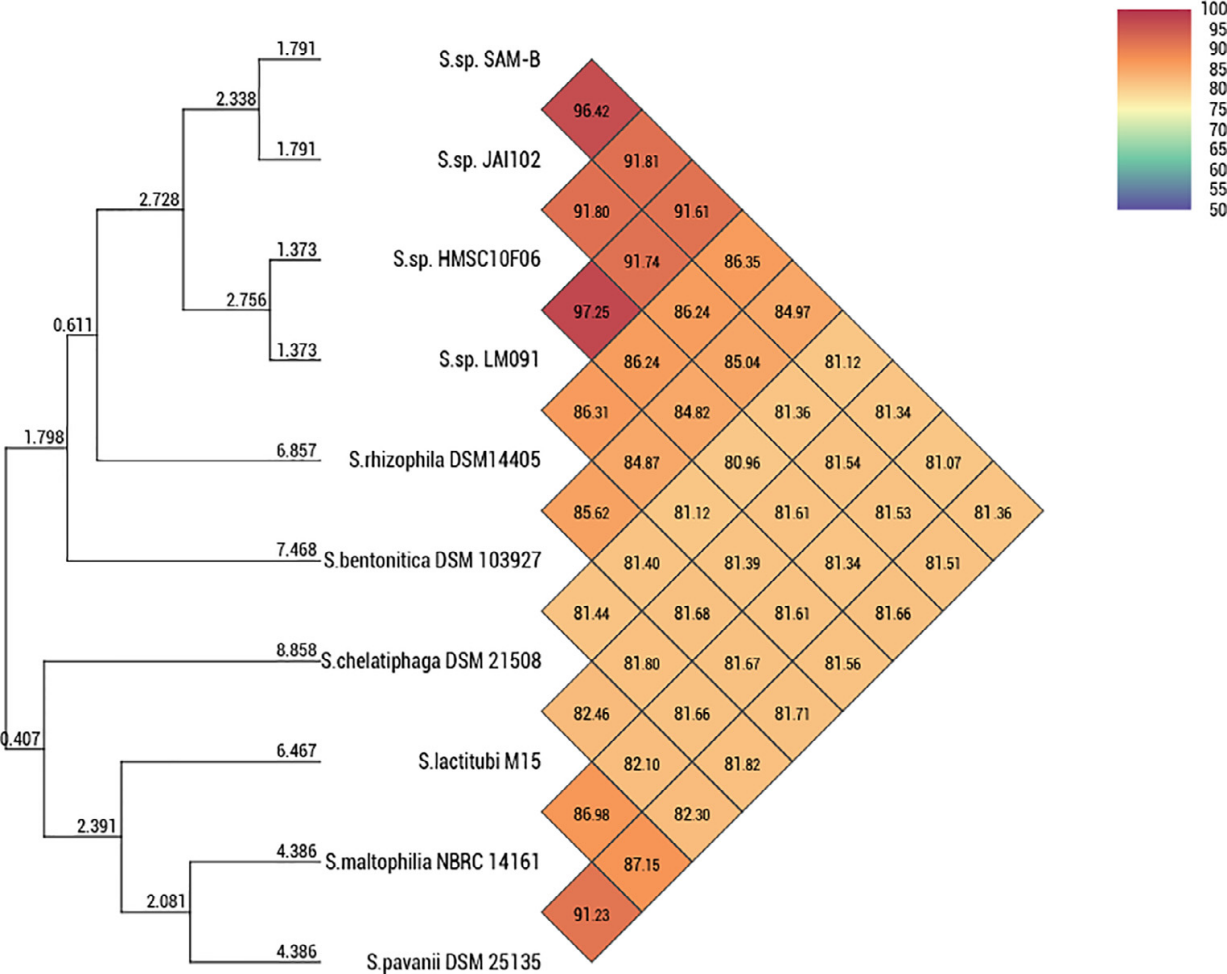
Heatmap generated according to OrthoANI values that were calculated by the OAT software for the genomes of the *Stenotrophomonas* sp. strain SAM-B and other closely-related members of the *Stenotrophomonas* genus.

According to the BLAST results with input data queried against the 16S ribosomal RNA (Bacteria and Archaea) NCBI database (query performed on 10.06.2020), the organism that is most similar to SAM-B strain according to homology that was determined through a query against the 16S rRNA gene (MT883430.1) was *Stenotrophomonas rhizophila* strain e-p10. The two most similar reference sequences NR_121739.1 and NR_028930.1 showed percent identities of 99.94% and 99.73%, respectively, whereas percent query covers were 99% and 96%, respectively. Other 16S rRNA reference sequences that were most similar to the SAM-B sequence and belonged to the *Stenotrophomonas* genus (NR_157765.1, NR_117406.1, NR_148818.1, and NR_116366.1), demonstrated lower percent identities (98.14–98.83%) and percent query covers (93%–95%). To achieve precise taxonomic assignment, we queried the genome of the SAM-B strain against the genomes from the RefSeq Genome Database (NCBI). Ten *Stenotrophomonas* genomes, which demonstrated the highest similarity according to 16S rRNA gene (≥99%) or genome pairwise comparison calculated by the Type (Strain) Genome Server (TYGS) [5], were selected for comparison in the OrthoANI test [6]. We used the genomes of three *Stenotrophomonas* spp. strains with no specific identification at the species level: LM091 (NZ_CP017483.1), JAI102 (NZ_JACCCI000000000.1), and HMSC10F06 (NZ_LWNH00000000.1); we also used the genomes of *Stenotrophomonas* spp. that belonged to six species, namely *S. rhizophila* DSM 14405 (CP007597.1), *S. bentonitica* DSM 103927 (NZ_JAAZUH000000000.1), *S. chelatiphaga* DSM 21508 (NZ_LDJK00000000.1), *S. lactitubi* M15 (PHQX00000000.1), *S. maltophilia* NBRC 14161 (BCUI00000000.1), and *S. pavanii* DSM 25135 (NZ_LDJN00000000.1). The genome of *Stenotrophomonas* sp. JAI102 was the most similar (96.42%) to that of the SAM-B strain according to the OrthoANI test. The OrthoANI values were calculated based on the pairs of genomes of the SAM-B strain and other strains or species; OrthoANI values that ranged from 81.07% to 86.35% were below the species boundary value (ANI, >95–96%) (Fig. 3). Thus, the results of the taxonomic assignment, based on queries against 16S rRNA genes and genomes (OrthoANI test), supported the assignment of the SAM-B strain under the genus *Stenotrophomonas*. This finding confirms the probable assignment of strain SAM-B to the undescribed species of *Stenotrophomonas* and provides insight for future research of diverse proteolytic bacteria in cold water environments that are yet to be discovered.

## 2 Experimental Design, Materials, and Methods

### 2.1 Sample collection and screening

A 1.5-L water sample from Uzharlyg Mineral Cold Spring (50.6158N, 94.9610 E), Samagaltay Settlement, Republic of Tyva (Southern Siberia), Russian Federation was collected and stored in sterile plastic container. The water temperature during the collection was 10°С. Psychrophilic bacteria were isolated in a plate with Pfennig medium (0.3 g/L KH_2_PO_4_; 0.3 g/L MgCl_2_ × 2H_2_O; 0.3 g/L NH_4_Cl; 0.3 g/L CaCl_2_; 0.5 g/L peptone; 30.0 g/L agar; pH 8). The plates were incubated at 10 °C and monitored for growth. The colonies that grew were subcultured several times on fresh media.

### 2.2 Genomic DNA extraction

A colony from a culture plate of the strain SAM-B was inoculated into a 5-ml Luria-Bertani medium and incubated overnight. Genomic DNA was extracted using a Quick-gDNA™ Mini Prep Kit (Zymo Research, USA). The quality of the extracted DNA was assessed according to A260/280 ratio using Nanodrop 8000 (Thermo Fisher Scientific, USA), and electrophoresis was performed in 1% agarose gel. DNA concentration was quantified by using Qubit 4.0 Fluorometer and a dsDNA High Sensitivity Assay Kit (Life Technologies, USA).

### 2.3 Library construction and genome sequencing

DNA library for the whole-genome sequencing was prepared using a NEBNext® Ultra™ II FS DNA Library Prep Kit for Illumina® (New England BioLabs, USA). Paired-end sequencing (2 × 300 bp) was carried out on a MiSeq platform (Illumina, USA) using a Reagent Kit v.3 (Illumina, USA) in the Center of Shared Scientific Equipment “Persistence of microorganisms” of the Institute for Cellular and Intracellular Symbiosis UrB RAS.

### 2.4 Bioinformatics treatment, genome annotation, and phylogenomic comparison

The quality of raw reads was assessed by using FastQC (version 0.11.7.0). The reads with ambiguous nucleotides, Illumina adapters, and low-quality reads were removed using Trimmomatic (version 0.36) [7]. *De novo* assembly was performed for several datasets with different trimming parameters using SPAdes v. 3.14.0 [8]. The assemblies were assessed using Quast (version 5.0.2) [9], and the best resulting variant was selected for annotation. Ribosomal RNA genes in the assembly were predicted using Barrnap (version 0.9). The final genome assembly was annotated using RAST [10] and NCBI Prokaryotic Genome Automatic Annotation Pipeline (PGAAP) [11]. The average nucleotide identity with reference to closely related genomes was determined using the Orthologous Average Nucleotide Identity Software Tool (OAT) [6].

## CRediT Author Statement

**Elena S. Kashkak:** Investigation, Writing - Original Draft, Funding acquisition. **Vladimir Ya. Kataev:** Investigation, Validation, Writing - Original Draft. **Yuri A. Khlopko:** Software, Formal analysis, Data Curation. **Valentina G. Budagaeva:** Investigation. **Erzhena V. Danilova:** Supervision, Resources. **Urana S. Oorzhak:** Sampling. **Olga P. Dagurova:** Resources. **Andrey O. Plotnikov:** Methodology, Writing - Original Draft, Writing - Review & Editing

## Ethical Statement

All ethical requirements were observed in the preparation of the publication. The work was not related to the use of human objects, and did not include experiments with animals.

## Declaration of Competing Interest

The authors declare that they have no known competing financial interests or personal relationships that could have appeared to influence the work reported in this paper.

## Appendix Supplementary materials

Image, application 1

Image, application 2

## Footnotes

Supplementary material associated with this article can be found, in the online version, at doi:10.1016/j.dib.2020.106278.
